# Trimethyl chitosan: Antibacterial activity on *Enterococcus faecalis* biofilm and cytocompatibility on human periodontal ligament fibroblasts cells

**DOI:** 10.1016/j.jds.2025.06.022

**Published:** 2025-07-15

**Authors:** Raras Ajeng Enggardipta, Minato Akizuki, Mika Bando, Yuji Inagaki, Kazumitsu Sekine, Kenichi Hamada, Tomoko Sumitomo, Kanta Sato, Hiromichi Yumoto

**Affiliations:** a Department of Periodontology and Endodontology, Tokushima University Graduate School of Oral Sciences, Tokushima, Japan; b Department of Conservative Dentistry, Faculty of Dentistry, Universitas Gadjah Mada, Yogyakarta, Indonesia; c Department of Periodontology and Endodontology, Tokushima University Graduate School of Biomedical Sciences, Tokushima, Japan; d Department of Biomaterials and Bioengineering, Tokushima University Graduate School of Biomedical Sciences, Tokushima, Japan; e Department of Oral Microbiology, Tokushima University Graduate School of Biomedical Sciences, Tokushima, Japan

**Keywords:** Biofilm, Cytocompatibility, *Enterococcus faecalis*, Trimethyl chitosan

## Abstract

**Background/purpose:**

Effective disinfection of the root canal system remains a major challenge due to complex anatomy and the persistence of biofilm-forming bacteria such as *Enterococcus faecalis*. Trimethyl chitosan (TMC), a quaternized chitosan derivative, has shown promising antimicrobial properties but has not been extensively studied for endodontic use. This study aimed to evaluate the antibacterial and antibiofilm efficacy of TMC against *E. faecalis* and assess its cytocompatibility with human periodontal ligament fibroblasts (HPdLFs).

**Materials and methods:**

The *E. faecalis* biofilm formation in the presence of TMC and antibacterial activity of TMC against mature *E. faecalis* biofilms were evaluated using crystal violet staining, adenosine triphosphate assays, colony forming unit counting, scanning electron microscopy, and fluorescence microscopy. The expression of genes associated with *E. faecalis* biofilm formation, such as *ace*, *esp*, and *gelE,* was determined. Moreover, cytocompatibility of TMC with HPdLFs was assessed using a cell counting kit-8 assay.

**Results:**

TMC significantly inhibited biofilm formation by *E. faecalis* and in the mature *E. faecalis* biofilm, TMC interfered with the total biofilm biomass, reduced bacterial numbers, weakened the biofilm structure, and upregulated *ace*, *esp*, and *gelE* expression. Furthermore, a lower concentration of TMC maintained HPdLFs viability.

**Conclusion:**

This study highlights the potential of TMC as a novel irrigating material owing to its antibacterial and antibiofilm activities against *E. faecalis* and its cytocompatibility with HPdLFs.

## Introduction

Eradicating bacteria and their by-products in the root canal system is a critical challenge for successful endodontic treatment, considering the persistence of root canal infections and post-treatment apical periodontitis that may arise from the failure of bacterial elimination.^1^ Despite advances in instrumentation, complete bacterial elimination remains a major challenge due to the complex anatomy of the root canal system and the presence of biofilm-forming microorganisms.^2^^,^^3^ Chemical irrigation plays a critical role in complementing mechanical cleaning by targeting residual bacteria and biofilms that instrumentation alone cannot remove.^4^^,^^5^

Sodium hypochlorite (NaOCl) is the commonly used endodontic irrigant due to its exceptional antimicrobial activity and tissue-dissolving capabilities. However, its cytotoxicity to periapical tissues, unpleasant taste, reduction in the mechanical properties of dentin, and inability to remove smear layer have raised concern.^4^^,^^6^ Other irrigants, such as chlorhexidine (CHX), ethylenediaminetetraacetic acid (EDTA), and more recently herbal or nanoparticle-base agents, have been investigated to improve safety and efficacy. Nevertheless, no single irrigant has yet demonstrated the ideal combination of strong antimicrobial activity, biofilm disruption, biocompatibility, and smear layer removal.^6^^,^^7^ Thus, the development of novel or adjunctive irrigants that can address these shortcomings continues to be explored.

*Enterococcus faecalis* is one of the top ten microorganisms most frequently found in persistent endodontic infections and obturated root canals with chronic apical periodontitis after endodontic treatment.^1^^,^^8^ Its ability to penetrate dentinal tubules, adapt to alkaline environments, and form biofilms contributes significantly to root canal treatment failure.^9^^,^^10^ For this reason, *E. faecalis* has become a widely accepted model organism for evaluating the antibacterial efficacy of new endodontic materials. While multispecies biofilm models more closely mimic clinical infections, single-species *E. faecalis* biofilms remain a valuable and relevant system, particularly for early-phase screening of novel therapeutic agents under controlled conditions.^11^^,^^12^

Chitosan-based compounds have drawn attention due to their inherent antimicrobial properties and compatibility with biological tissues.^13^^,^^14^ Trimethyl chitosan (TMC), a quaternized derivative of chitosan, offers improved solubility in neutral and alkaline pH, enhanced permeability, and broad-spectrum antimicrobial activity.^15^^,^^16^ TMC has been extensively studied in biomedical applications.^15^ However, its potential as an endodontic irrigant remains underexplored. This study aimed to evaluate the antibacterial activity of TMC against *E. faecalis* biofilm and assess its cytocompatibility with human periodontal ligament fibroblasts (HPdLFs). The findings are expected to contribute to the development of irrigation materials addressing the need for innovative disinfection strategies that can improve treatment outcomes.

## Materials and methods

### Preparation of trimethyl chitosan

TMC (Sigma–Aldrich, St. Louis, MO, USA) was dissolved in distilled water at concentrations of 1, 3, and 5 mg/mL, which were sub-inhibitory concentrations for *E. faecalis* ATCC19433 (OD_550_ = 0.1), based on the preliminary screening using a broth microdilution technique (CLSI M07 guidelines) with adaptation for chitosan derivatives.^17^^,^^18^

### Effect of trimethyl chitosan on planktonic *E. faecalis*

*E*. *faecalis* ATCC19433 (OD_550_ = 0.1) was dispensed into 96-well microplate (100 μL/well) and treated with TMC at final concentrations of 1, 3, and 5 mg/mL. Untreated wells served as control. Plate was incubated anaerobically at 37 °C for 24 h. For these analyses, different sets of wells within the same microplate were assigned to crystal violet (CV) staining or adenosine triphosphate (ATP) assay. The biofilms formed in the wells were quantified using a CV staining.^19^ Briefly, biofilm was stained with 0.1 % (w/v) CV (200 μL/well; TCI, Tokyo, Japan) for 15 min at room temperature (RT; 20–25 °C), washed twice with phosphate-buffered saline (PBS; Nissui, Tokyo, Japan), and dried for 15 min. Subsequently, 99.9 % ethanol (200 μL/well; Wako, Osaka, Japan) was used to solubilize the biofilm for 10 min. After that, the absorbance was measured with a microplate reader (iMark; Bio-Rad, Tokyo, Japan) at 560 nm.

The BacTiter-Glo™ adenosine triphosphate (ATP) assay (Promega Corp., Madison, WI, USA) was employed to evaluate metabolic activity of *E. faecalis*. After removing the culture medium containing planktonic bacteria and washing with PBS to remove unattached bacteria, the reagent and PBS (total 200 μL/well) were mixed and incubated for 5 min in the dark at RT. Subsequently, the solution (150 μL/well) was transferred to an opaque-walled microplate and luminescence was measured using a microplate reader (Infinite M200 Pro; TECAN, Grödig, Austria).

### Effect of trimethyl chitosan on *E*. *faecalis* biofilm

Biofilms were formed on the upper surface of sterilized hydroxyapatite (HA) pellets (10 mm^2^, 2 mm in thickness; HOYA Technological Co., Tokyo, Japan). HA pellets were placed on the bottom of a 24-well plate, filled with 500 μL of *E. faecalis* (OD_550_ = 0.1) and anaerobically incubated at 37 °C for two weeks. The culture medium was replenished every 48 h.

After two weeks of biofilm formation, the HA pellets were rinsed with PBS and immersed in TMC (1, 3, and 5 mg/mL) for 30 s. Subsequently, 2.5 % sodium hypochlorite (NaOCl; Neo Dental Chemical Products Co. Ltd., Chiba, Japan) and PBS were used as positive and negative controls, respectively. The HA pellets then were washed twice with PBS and their antibacterial activity was assessed.

CV staining was performed to evaluate the biofilm biomass on the surface of HA pellets. The HA specimens were immersed in 500 μL of 0.1 % CV for 10 min at RT, rinsed, air-dried and solubilized in 99.9 % ethanol for 10 min. Subsequently, 100 μL of decolorized solutions were transferred into a 96-well plate and measured the optical density (OD) at 560 nm.

A scanning electron microscopy (SEM) method was used to analyze the biofilms’ morphology.^20^^,^^21^ HA pellets were fixed in 1 mL of 2.5 % (v/v) glutaraldehyde (TAAB Laboratories, Berks, England) for 10 min; serially dehydrated using 40 %, 75 %, and 99.9 % ethanol; and air-dried at RT. After gold-sputtering, the HA pellets were observed under a SEM (JCM-5700; JEOL, Peabody, MA, USA).

The biofilms formed on the HA pellets was mechanically removed using a shaker (TAITEC Invitrogen Shaker, Saitama, Japan) and cell scrapper. The biofilm suspension was then used for colony forming unit (CFU) counting and ATP assay. The biofilm suspension was serially diluted, plated onto BHI agar using an automatic spiral plater (EasySpiral; Interscience, Tokyo, Japan) and anaerobically incubated at 37 °C for 48 h. Subsequently, colonies were counted, and CFUs/mL were calculated. An ATP assay (BacTiter-Glo™, Promega Corp.) was performed according to the manufacturer's protocol to evaluate *E. faecalis* metabolic activity in biofilms. The biofilm suspension (100 μL) from each sample was added to an opaque-walled 96-well plate. Then, 100 μL of the BacTiter-Glo™ reagent (Promega Corp.) was added to each well. After 5-min of incubation, luminescence was measured using a microplate reader (Infinite M200 Pro; TECAN).

For live/dead analysis, *E. faecalis* biofilms were grown on glass-bottom dish (∅35 mm; Violamo AS One Corporation, Osaka, Japan) for two weeks. Biofilms were treated with TMC (1, 3, and 5 mg/mL) for 30 s, rinsed with filter-sterilized water, and stained using the FilmTracer™ LIVE/DEAD Biofilm Viability Kit (Thermo Fisher Scientific, Waltham, MA, USA) for 30 min in the dark. Fluorescence images were taken at five random points under a fluorescence microscope (BZ-X800; KEYENCE, Osaka, Japan), and live/dead cells were quantified following the protocol for image analysis using Fiji ImageJ software version 2.14.0/1.54f.^22^

The biofilm suspensions were centrifuged (1,000×*g*, 10 min), and the bacterial cell pellets were lysed using lysozyme (Thermo Fisher Scientific). Total RNA was extracted using the Nucleospin RNA Isolation Kit (Bioanalysis Macherey–Nagel, Duren, Germany) and reverse transcribed into cDNA with PrimeScript™ Reverse Transcriptase Kit (Takara Bio, Osaka, Japan). Real-time PCR was performed using the SYBR Green Supermix (Bio-Rad) on a CFX96™ Real-Time System (Bio-Rad), with *16S* rRNA as the reference gene. The cycling condition consisted of 95 °C for 2 min and 40 repeats of the following steps: 95 °C for 30 s, 60 °C for 1 min, and 72 °C for 1 min. Gene expression was analyzed using the 2^−ΔΔ*CT*^ method and primers sequences are listed in Table 1.

**Table 1:** The sequences of primers for qRT-PCR.

Name		Sequence (5ʹ-3ʹ)	Amplicon size (base pairs)	Reference
*16S* rRNA^a^	F	CCCAACATCTCACGACACGA	202	This study
	R	GAGTACGACCGCAAGGTTGA		
*ace*	F	AGCGGCAATCAAAATGTGGA	111	24
	R	TGTTTCTGTTGCCTGTTCCG		
*esp*	F	AATGATTCGCTTGGCAGACC	105	24
	R	AGGAGCAGTTTGTGTATCAGTTG		
*gelE*	F	CGGATTGGTTACACCATTATCC	296	25
	R	TGCCACTCCTTATCCATTTTT		

qRT-PCR, quantitative reverse transcription polymerase chain reaction. *rRNA*, ribosomal ribonucleic acid. *ace*, adhesin to collagen from *Enterococcus faecalis*. *esp*, enterococcal surface protein. *gelE*, *Enterococcus faecalis* gelatinase.

a Primers were designed for this study using the Primer-BLAST NCBI website (https://www.ncbi.nlm.nih.gov/tools/primer-blast/).

### Cytocompatibility of trimethyl chitosan on human periodontal ligament fibroblasts

Cytotoxicity assay was assessed by Cell Counting Kit-8 (CCK-8; Dojindo Laboratories, Kumamoto, Japan) assay according to manufacturer's protocol and the ISO 10993–5:2009(e). HPdLFs (primary cells; Lonza Bioscience, Tokyo, Japan) was seeded into a 96-well plate (1 × 10^4^ cells/100 μL/well) and incubated for 24 h (37 °C, 5 % CO_2_). TMC at final concentration of 1, 3, and 5 mg/mL, and 2.5 % NaOCl were added into the well, followed by incubation for 5 min, simulating root canal preparation.^23^ After removing the culture medium, a fresh culture medium (100 μL/well) was added to each well, and the cells were continued to be incubated for up to 72 h. At the time points of 0 h, 24 h, 48 h, and 72 h after incubation times, Cell Counting Kit-8 (CCK-8) solution (Dojindo Laboratories) was added to each well (10 μL/well) and incubated for 2 h. The absorbance was measured at 450 nm. The cell viability was calculated by the following formula: the %viability = [(ODsample – ODblank)/(ODcontrol – ODblank)] × 100 %.

### Statistical analyses

Normality and homogeneity of variance tests were performed for all datasets. Depending on the results, appropriate statistical tests were applied: one-way ANOVA with Bonferroni or Games-Howell post-hoc test, or Kruskal–Wallis with Mann–Whitney post-hoc tests. Two-way repeated-measures ANOVA with a simple effect test was used for the cytocompatibility data. Analyses were conducted using IBM SPSS Statistics v29 (IBM; Chicago, IL, USA), with significance level of *P* < 0.05.

## Results

### Effect of trimethyl chitosan on planktonic *E. faecalis*

**Figure 1:**
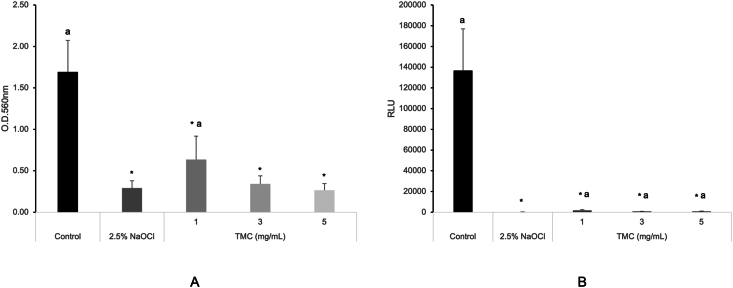
Inhibitory effects of TMC on biofilm formation and metabolic activity of *E. faecalis* during 24-h biofilm formation, analyzed by CV staining (A) and ATP in vitro (B). Asterisks and “a” denote a statistically significant difference compared to the negative control (PBS) and the positive control (2.5 % NaOCl), respectively (*P* < 0.05). ATP, adenosine triphosphate; CV, crystal violet; NaOCl, sodium hypochlorite; PBS, phosphate-buffered saline; TMC, trimethyl chitosan.

All tested concentration of TMC showed lower biofilm biomass (OD value) and metabolic activity (RLU) of *E. faecalis* during the 24-h biofilm formation compared to the PBS group (*P* < 0.05), as determined by the Kruskal–Wallis test followed by Mann–Whitney post hoc analysis. However, 2.5 % NaOCl showed stronger effects than TMC (Fig. 1A and B).

### Effect of trimethyl chitosan on *E. faecalis* biofilm

**Figure 2:**
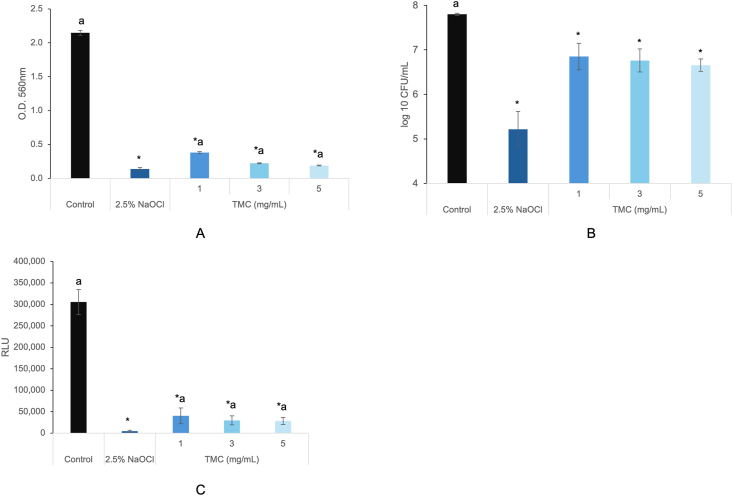
Antibacterial activity of TMC against *E. faecalis* biofilms on HA pellets. The antibacterial effects of TMC on 2-week-old *E. faecalis* biofilm were analyzed by (A) CV staining, (B) colony-forming unit (CFU) counting, and (C) ATP assay in vitro. Asterisks and “a” denote a statistically significant difference compared to the negative control (PBS) and the positive control (2.5 % NaOCl), respectively (*P* < 0.05). ATP, adenosine triphosphate; CV, crystal violet; HA, hydroxyapatite; NaOCl, sodium hypochlorite; PBS, phosphate-buffered saline; TMC, trimethyl chitosan.

**Figure 3:**
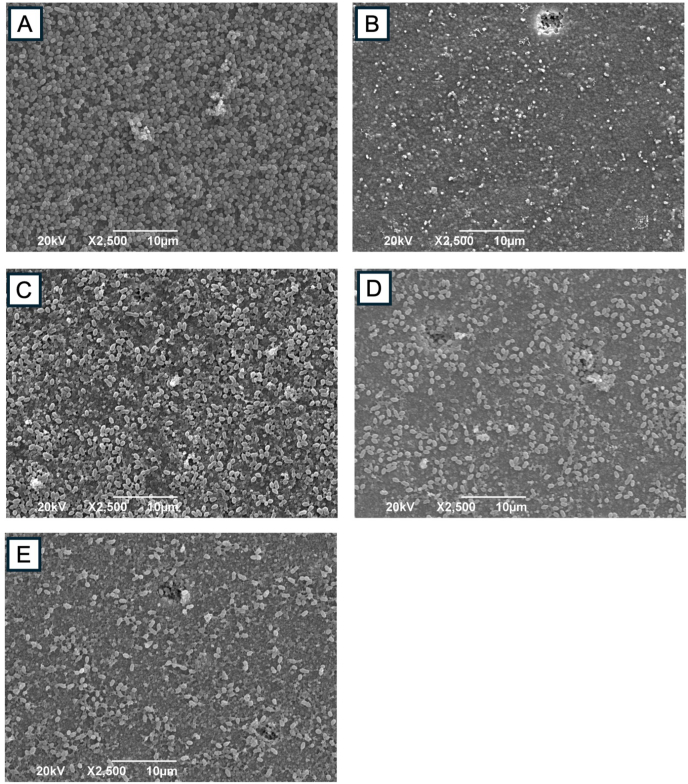
Disruption of *E. faecalis* biofilm by TMC treatment. Scanning electron microscope (SEM) images shows the morphology of 2-week-old *E. faecalis* biofilms on the HA pellet surfaces after 30-s treatment with the different concentrations of TMC ( × 2,500; 20 kV). Panels show representative biofilm images of *E. faecalis* in the negative control group (PBS: A), positive control group (2.5 % NaOCl: B), 1 mg/mL (C), 3 mg/mL (D), and 5 mg/mL (E) of TMC, respectively. HA, hydroxyapatite; NaOCl, sodium hypochlorite; PBS, phosphate-buffered saline; TMC, trimethyl chitosan.

**Figure 4:**
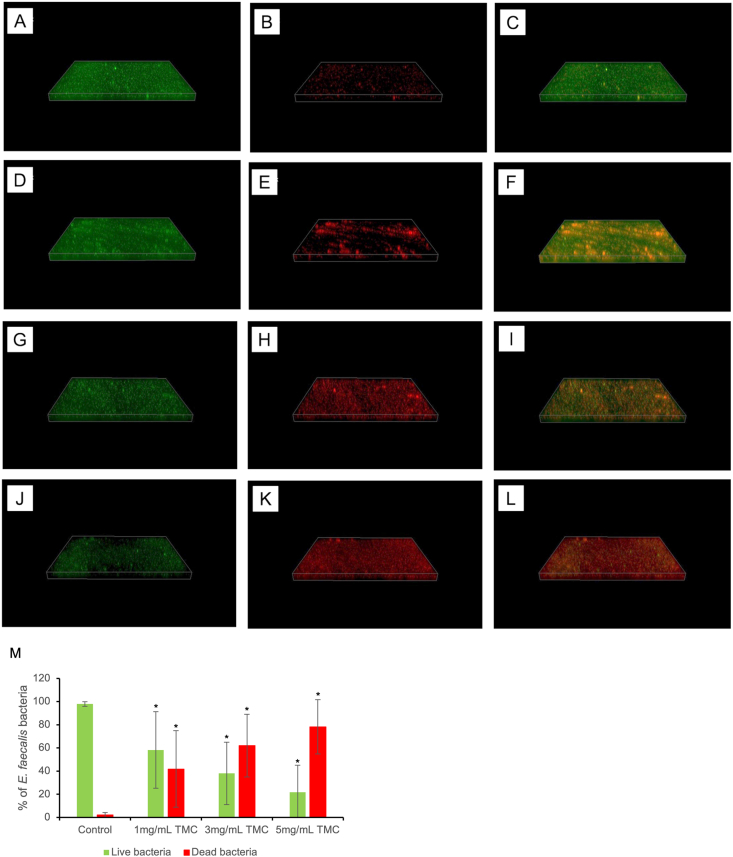
Biofilm analysis using fluorescence microscope. (A–C) negative control, (D–F) 1 mg/mL TMC-treated group, (G–I) 3 mg/mL TMC-treated group, (J–L) 5 mg/mL TMC-treated group. Green fluorescence represents viable *E. faecalis* cells, while red fluorescence indicates dead cells. (M) The quantification of bacteria viability was performed using Fiji ImageJ software. Results were the average of five randomly selected areas of each sample and are presented as mean ± standard deviation (∗*P* < 0.05 vs negative control group (PBS), Kruskal–Wallis test with Mann–Whitney U Post Hoc test). The 2.5 % NaOCl as positive control group was omitted due to complete biofilm removal on the glass-bottom dish. NaOCl, sodium hypochlorite; PBS, phosphate-buffered saline; TMC, trimethyl chitosan.

**Figure 5:**
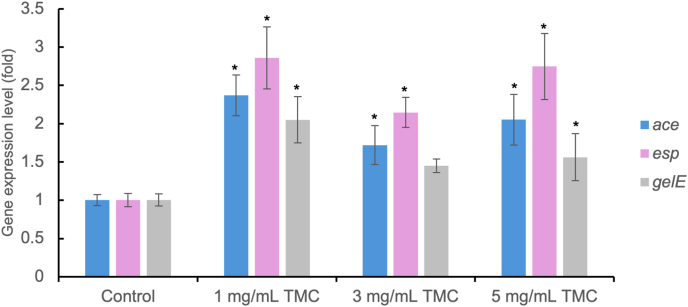
Regulation of *E. faecalis* biofilm-related gene expression by TMC. The expression levels of biofilm-related genes were determined by qRT-PCR. The 16S rRNA gene was used as internal control. The values are represented as means ± standard deviation from quadruplicate samples. Data are representative of at least three independent experiments. (∗*P* < 0.05 vs negative control group, one-way ANOVA with Bonferroni Post-Hoc test). qRT-PCR, quantitative reverse transcription polymerase chain reaction; rRNA, ribosomal ribonucleic acid; TMC, trimethyl chitosan.

CV staining (Fig. 2A) showed that TMC (1, 3, 5 mg/mL) significantly decreased biofilm biomass compared to PBS (*P* < 0.05), as determined by the Kruskal–Wallis test with Mann–Whitney post hoc analysis. CFU analysis (Fig. 2B) demonstrated reduced bacterial viability in TMC-treated groups, with statistical significance confirmed by one-way ANOVA followed by Games-Howell post hoc test (*P* < 0.05). Similarly, ATP assay (Fig. 2C) confirmed a significant reduction in metabolic activity in TMC-treated groups compared to PBS (*P* < 0.05), based on the Kruskal–Wallis test and Mann–Whitney post hoc analysis. SEM images revealed dense biofilm in the PBS group (Fig. 3A), sparse bacterial presence with 2.5 % NaOCl (Fig. 3B), and visibly disrupted biofilm in all TMC-treated groups (Fig. 3C–E). Live/dead staining showed TMC treatment decreased viable cells (green fluorescence; Fig. 4D–G) and increased dead cells (red fluorescence; Fig. 4E–K). Quantitative analysis confirmed a significant reduction in viability in TMC-treated biofilms (*P* < 0.05; Fig. 4M). These differences were statistically significant according to the Kruskal–Wallis test with Mann–Whitney post hoc analysis. qRT-PCR showed that TMC significantly upregulated *E. faecalis* biofilm related genes (*ace*, *esp*, *gelE*) at all tested concentrations, with the highest expression observed at 1 mg/mL (*ace*: 2.3-fold; *esp*: 2.8-fold; *gelE*: 2-fold; *P* < 0.05), as analyzed by one-way ANOVA with Bonferroni post hoc test (Fig. 5).

### Cytocompatibility of trimethyl chitosan on human periodontal ligament fibroblasts

**Figure 6:**
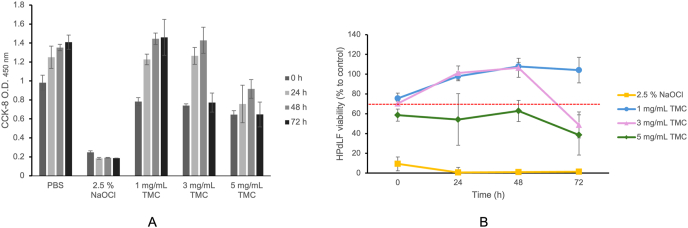
Cytocompatibility of TMC on HPdLFs. Optical density value (A) and percentage of HPdLFs viability (B) after exposure to different concentrations of TMC (1, 3, and 5 mg/mL) for 5 min were quantified using Cell Counting Kit-8 (CCK-8) assay at 0, 24, 48, and 72 h. The percentage of HPdLFs viability in the experimental groups is calculated according to the absorbance of the negative control group, which is set to 100 %. The red-dash line at 70 % represents the 30 % reduction threshold per the ISO 10993–5:2009(e) recommendation, considering values below this line to be cytotoxic. Data are representative of at least three independent experiments. HPdLFs, human periodontal ligament fibroblasts; TMC, trimethyl chitosan.

TMC-treated groups showed significantly higher cytocompatibility with HPdLFs than 2.5 % NaOCl (Fig. 6A). Based on the 30 % reduction threshold ISO 10993–5:2009(E) recommendation, 1 mg/mL TMC was cytocompatible throughout the incubation period, 3 mg/mL remained cytocompatible up to 48 h, and 5 mg/mL stayed below the threshold over time (Fig. 6B). Two-way repeated-measures ANOVA revealed a significant interaction between treatment groups and observation time (*P* = 0.039). Simple-effect testing showed no significant difference between TMC-treated groups and PBS (*P* > 0.05), confirming TMC's markedly lower cytotoxicity compared with 2.5 % NaOCl.

## Discussion

TMC is a water-soluble cationic chitosan derivative with stable positive charge across pH levels, retaining its biocompatibility while enhancing antimicrobial activity, solubility, and mucoadhesive capacity. Its bacterial effect is up to 700 times stronger than pure chitosan due to the presence of ^-+^N(CH3)_3_ quaternary groups.^26^ In this study, TMC significantly reduced biofilm biomass and bacterial metabolic activity during 24 h of biofilm formation, indicating effective elimination of *E. faecalis* planktonic bacteria. This supports the strategy of inhibiting biofilm development by targeting planktonic bacteria early in the infection process.^27^

TMC demonstrated antibacterial activity against 2-week-old *E. faecalis* biofilms, significantly reducing biofilm biomass, colony counts, and metabolic activity compared to PBS. SEM analysis confirmed visible biofilm disruption in the TMC-treated groups, compared to the dense biofilm observed in the PBS group. These findings support the antibacterial activity of TMC against *E. faecalis* biofilms. *E. faecalis* is a Gram-positive bacteria with a cell wall structure composed of peptidoglycan, wall teichoic acids (WTAs) covalently attached to the peptidoglycan layer, and lipoteichoic acids (LTAs) connected to the cell membrane. Both WTAs and LTAs have negatively charged anionic backbones.^28^ Therefore, the cationic TMC polymer has a high-positive-charge density, which enables it to interact with negatively charged bacterial cell walls. This interaction occurs between the cationic N-quaternized groups (^-+^N(CH3)_3_) on TMC and anionic molecules, such as phospholipid dipalmitoyl phosphatidylglycerol (PDPPG), WTAs, and LTAs on bacterial cells.^28^, ^29^, ^30^ As a result, membrane permeability significantly increases, leading to structural alterations (distortion and disruption), severe leakage of cytoplasmic contents, and, ultimately, bacterial cell death.^30^

This study suggests that the interaction between TMC and *E. faecalis* leads to the destruction of the bacterial cell membrane. This was supported by the results of fluorescence microscopy using two types of dyes, indicating cell viability based on membrane permeability. SYTO 9 and propidium iodide (PI) fluoresce when bound to nucleic acids. However, PI cannot penetrate intact cell membranes and, therefore, does not stain live cells, whereas SYTO 9 can freely enter and stain living cells.^31^ Fluorescence microscopy images after TMC treatment clearly showed an increase in the percentage of dead cells (red fluorescence signal) in the biofilm.

HPdLFs are the primary cells that respond to substances applied endodontically to periapical tissues when irrigants accidently exit the apical foramen. Irrigation that leaks through the apical foramen during root canal treatment can cause inflammation in the surrounding periapical tissue and suppress tissue healing and regeneration.^32^^,^^33^ The concentration of NaOCl generally used in clinics ranges from 0.5 to 8.25 %, all of which possess antibacterial properties. The higher the concentration, volume, contact time, and temperature of NaOCl, the higher the bactericidal effect and tissue dissolution ability. Simultaneously, the irritation, causticity, and cytotoxicity to tissues are also increasing.^4^^,^^34^^,^^35^ Therefore, although 2.5 % NaOCl showed potent antibacterial activity in this study, its high cytotoxicity must be considered. This study revealed that 5 min exposure of 2.5 % NaOCl resulted in the lowest percentage of HPdLFs viability compared with that of TMC-treated groups. An exposure period of 3–10 min is considered appropriate to simulate the transient contact between endodontic irrigants and periradicular tissues, such as the periodontal ligament, dental pulp, and apical papilla, during routine chemo-mechanical root canal procedures.^23^

Biofilm formation in *E. faecalis* involves multiple genes, including *ace*, *esp*, and *gelE*, which contribute to adhesion, colonization, and persistence during infection.^25^^,^^36^^,^^37^ In this study, treatment with TMC upregulated the expression of *ace*, *esp*, and *gelE*, indicating a stress response by surviving bacteria on the HA pellets surface. This result aligns with previous findings where sub-inhibitory concentrations of antimicrobials or environmental stressors induced similar genes expression changes as part of bacterial adaptive responses.^38^, ^39^, ^40^ Notably, the highest gene expression was observed at the 1 mg/mL of TMC, despite a brief exposure time (30 s). This suggests that while TMC is antibacterial, short or suboptimal exposure may trigger transient stress response in residual bacteria. Therefore, further studies are needed to assess whether prolonged or optimized exposure can suppress this upregulation and prevent potential risks such as increased virulence or reinfection.

It is well recognized that there is no universally accepted standard for endodontic irrigation procedures.^34^ In clinical endodontics, irrigation procedures may vary depending on the operator preferences or clinical scenario. Previous studies showed wide variability in root canal irrigation parameters, such as irrigants concentration, contact time and delivery method. It is generally accepted that longer exposure durations enhance antibacterial efficacy. However, reported exposure time was vary from seconds to 30 min depending on the specific experimental design and objectives.^4^^,^^7^^,^^41^^,^^42^ Therefore, the approach adopted in this study reflects an early-stage, controlled evaluation to investigate the antibacterial activity and cytocompatibility of TMC.

Based on the findings of the present study, TMC exhibited antibacterial and antibiofilm properties against *E. faecalis* while maintaining cytocompatibility with HPdLFs. Although TMC is less potent than 2.5 % NaOCl, its favorable cytocompatibility suggests potential as an alternative root canal irrigant. Considering that the effectiveness of root canal irrigants depends not only on their antibacterial strength but also on their biocompatibility with periapical tissues, TMC warrants further investigation. Further studies should focus on optimizing its formulation, concentration, and application protocol, including contact time, delivery systems, and possible synergistic combinations to enhance its clinical efficacy while maintaining tissue safety.

## Declaration of competing interest

The authors have no conflicts of interest relevant to this article.
