# Supplementary material for: Trimethyl chitosan: Antibacterial activity on Enterococcus faecalis biofilm and cytocompatibility on human periodontal ligament fibroblasts cells
Source: J Dent Sci. 2025 Jul 15;21(1):150–8. doi: 10.1016/j.jds.2025.06.022 (PMC12825504; doi:10.1016/j.jds.2025.06.022)
Supplement: Multimedia component 1 [file mmc1.docx]

**Supplementary Figures**

(a)

| 20 | 10 | 5 | 2.5 | 1.25 | 0.625 | 0.312 | 0.156 | 0.078 | sterile | (+) control | (-) control |
| --- | --- | --- | --- | --- | --- | --- | --- | --- | --- | --- | --- |


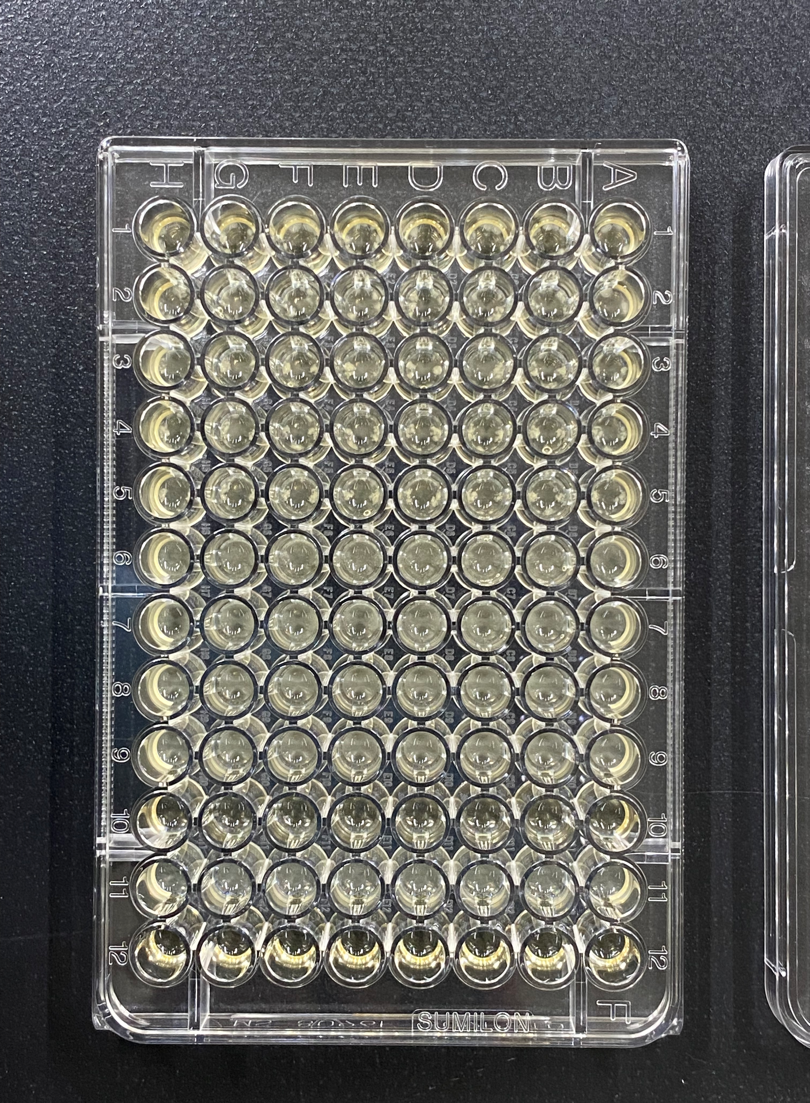


(b)

**Supplementary Figure 1** The minimal inhibitory concentration (MIC) of trimethyl chitosan (TMC). We used broth microdilution method based on the Clinical and Laboratory Standards Institute (CLSI M07) guidelines, with adaptations for chitosan derivatives. Serial two-fold dilutions of TMC in concentrations ranging from 20 mg/mL to 0.078 mg/mL with adjusted *E. faecalis* ATCC19433 concentration (OD_550_ = 0.1) were used to determine MIC in BHI broth. A positive control was bacterial inoculated, a negative control was only BHI broth, and only 0.078 mg/mL of TMC was served as sterile. Plates were incubated for 24 h at 37 °C. (A) The optical density value of all groups, (B) The 96-well microplate shows turbidity status in the wells. BHI, brain-heart infusion; MIC, minimal inhibitory concentration; OD, optical density; TMC, trimethyl chitosan.
